# Generation of HBV cccDNA using single-stranded M13 phage DNA for authentic minichromosome functionality

**DOI:** 10.1128/jvi.00035-26

**Published:** 2026-05-14

**Authors:** Yumeng Li, Ting Hua, Menghan Hao, Liman Chen, Wenjun Huang, Zhong Fang, Asha Ashuo, Yaming Li, Zhigang Yi, Hongzhou Gu, Zhenghong Yuan, Jieliang Chen

**Affiliations:** 1 MOE/NHC/CAMS Key Laboratory of Medical Molecular Virology, School of Basic Medical Sciences, Shanghai Institute of Infectious Disease and Biosecurity, Shanghai Medical College, Fudan University 58305, Shanghai, China; 2 Institutes of Biomedical Sciences, Fudan University, Shanghai, China; 3 Department of Chemical Biology, School of Chemistry and Chemical Engineering, and School of Global Health, Shanghai Jiao Tong University 596244, Shanghai, China; Wake Forest University School of Medicine, Winston-Salem, North Carolina, USA

**Keywords:** hepatitis B virus, covalently closed circular DNA, minichromosome, ssDNA, antivirals

## Abstract

Hepatitis B virus (HBV) covalently closed circular DNA (cccDNA) persists as minichromosomes in the nuclei of hepatocytes and serves as the transcriptional template for viral RNAs. The stable cccDNA pool, characterized by a very low copy number, poses challenges for studying its molecular characteristics and developing targeted therapeutics. To accurately mimic the full-length sequences and structure of HBV cccDNA, we developed a novel method using an M13 phage-based system and deoxyribozyme to produce full-length single-stranded DNA (ssDNA) corresponding to both the positive and negative strands of cccDNA. This method involves the cyclization of the ssDNA followed by the addition of T4 DNA ligase, thereby producing the cccDNA. The *in vitro* cyclized cccDNA, formed by M13 phage-derived single-stranded DNA (McccDNA), allows the formation of minichromosomes in transiently transfected hepatic cells, recapitulates key stages of the HBV life cycle, and effectively responds to multiple antiviral drugs. Additionally, McccDNA, as it contains no exogenous sequences, was shown to accurately transcribe major viral RNAs and organize nucleosomes in a sequence-specific manner. Furthermore, the McccDNA model allows for targeted deletion of viral proteins, producing a loss-of-function phenotype useful for further minichromosome studies. In conclusion, McccDNA generated from single-stranded M13 phage DNA recapitulates the natural properties of HBV cccDNA and serves as an authentic model to study cccDNA minichromosomes functionality.

**IMPORTANCE:**

HBV remains a major global health burden, with cccDNA serving as the transcriptional template for viral RNAs that sustain persistent infection. The low copy number of cccDNA in the nucleus and the lack of authentic *in vitro* models hinder its investigation and therapeutic development. Here, we established an M13 phage–derived single-stranded DNA approach to efficiently generate sequence-authentic HBV cccDNA *in vitro*. Upon transfection, the cccDNA molecules assembled into minichromosomes and were proven to be biologically active, producing viral RNAs more faithfully recapitulating infection-derived transcripts compared to recombinant cccDNA. The system also permits targeted mutagenesis for loss-of-function studies as well as site-specific labeling. These results provide insights into the nucleosome organization and transcriptional characteristics of the HBV cccDNA minichromosome, offering an authentic platform to investigate minichromosome functionality and host-virus interactions.

## INTRODUCTION

Hepatitis B virus (HBV) infection remains a severe global health problem. Despite the availability of preventive vaccines, approximately 296 million individuals worldwide are chronically infected with hepatitis B (1). Once chronic hepatitis B is established, failure to initiate timely intervention and treatment significantly increases the risk of progression to cirrhosis and hepatocellular carcinoma.

HBV is a sophisticated hepatotropic DNA virus that enters hepatocytes through the sodium taurocholate co-transporting polypeptide (NTCP) receptor (2, 3). After entry, the virus undergoes uncoating, releasing its relaxed circular DNA (rcDNA) genome into the nucleus (4, 5). Within the nucleus, the rcDNA is converted into covalently closed circular DNA (cccDNA) through the action of various host repair factors (6–9). The long-term persistence of cccDNA in the nuclei of infected hepatocytes poses significant challenges for its elimination. This is primarily due to its binding to host histones to form a stable minichromosome structure and its role as a template for HBV transcription and replication, which facilitates viral packaging and recycling (10, 11). Consequently, the enduring presence of HBV cccDNA is a critical obstacle in curing chronic hepatitis B. Notably, current clinical therapies do not specifically target cccDNA, underscoring a significant gap in the development of targeted therapeutics.

The exceptionally low copy number of HBV cccDNA presents a substantial challenge for conducting related research. In hepatocytes infected with HBV, the copy number of cccDNA is extremely low, with only 1–10 copies per cell, which significantly hampers the detection of cccDNA and the conduct of deeper study (12, 13). Recently, the recombinant cccDNA (rcccDNA) model reported by our group and others, which utilizes cyclization of the HBV genome via recombinase system, was shown to resemble cccDNA molecules and achieve an easily detectable level through transfection (14–18). However, due to the overlapping open reading frames in the HBV genome, the introduction of exogenous sequences during the process may disrupt the expression levels of multiple viral proteins, thereby impairing the replication activity. Consequently, there remains an urgent need for more authentic models to study the functionality of cccDNA minichromosomes.

In this study, we developed a novel approach to produce authentic cccDNA molecules (McccDNA) with relatively high efficiency using an M13-based ssDNA production system. We employed a single-stranded DNA annealing method to achieve cyclization, followed by ligation to generate DNA in a covalently closed circular form, identical to HBV cccDNA. *In vitro* cyclized McccDNA successfully assembled into a minichromosome, demonstrating robust antigen expression and replication, as well as significant responsiveness to multiple antiviral drugs targeting various stages of the HBV lifecycle. Additionally, McccDNA accurately transcribes major viral RNAs and organizes nucleosomes in a sequence-specific manner, similar to the natural infection system. In conclusion, McccDNA closely mimics the natural properties of HBV cccDNA, serving as a valuable model for advancing research on antiviral therapies and elucidating the molecular characteristics of cccDNA and antiviral responses during HBV infection.

## MATERIALS AND METHODS

### Cell lines, plasmids, and reagents

The HepG2-NTCP cell line was provided by Prof. Stephan Urban. The HepDE19 cell line, supplied by Prof. Jutao Guo, was used to produce HBV particles (13). Plasmids for the rcccDNA system were kindly provided by Prof. Deng Qiang. For the McccDNA system, plasmids were constructed by inserting the full-length HBV genome of genotype D (subtype ayw; GenBank accession no. V01460.1) into the p8000 vector, with Class-I and Class-II deoxyribozyme sequences flanking the insertion (19). The p8000 vector was derived from pBluescript, extended with a segment of λ phage DNA to reach a length of 8,000 bp. For the construction of p8000-phage-ccc(+), the HBV genome (nucleotides 1,574–1,573) was flanked by Class-I deoxyribozyme at the 5′ end (5′-ACGGTGGTTAGTTGAGCTGTCACGTCGAATCGACGTGACGTTGA-3′) and Class-II deoxyribozyme at the 3′ end (5′-AGCATCTTTGGCGATCAGCTAAGCTGATCGCTAGGGGAATAAATCTTTGGGCACCATGCGACG-3′). Similarly, for p8000-phage-ccc(−), the HBV genome (nucleotides 1,225–1,224)was flanked by Class-I deoxyribozyme at 5′ end (5′-GTTTTTGCTAGTTGAGCTGTCACGTCGAATCGACGTGACGTTGA-3′) and Class-II deoxyribozyme at the 3′ end (5′-AGCATCTTAGTAGCGATCAGCTAAGCTGATCGCGATTGGGGAATAGATCTTTGGGACTCTGACTTCTT-3′). The p8000-phage-ccc(−)_gap was truncated by 24 bp (nucleotides 1,225–1,248) compared to p8000-phage-ccc(−), and the biotin dT-oligo (5′-AGGCAAAAACGAGAG/Biotin-dT/AACTCCAC, GenScript Biotech.) was used to restore the gap. The single-site mutation at the first ATG codon within the HBV core (HBc) protein open reading frame (ORF) was altered to ATA to form HBc-deficient McccDNA model, and the eighth codon CAA in HBx ORF was mutated to TAA to form HBx-deficient McccDNA model. The siRNA used in this study was synthesized by Guangzhou RiboBio Co., Ltd with the sequences described previously (20, 21). All the small molecular compounds were purchased from MedChemExpress. Recombinant interferon alpha subtypes were obtained from PBL Assay Science (Piscataway, NJ). ELISA kits for detecting HBsAg and HBeAg were purchased from AutoBio.

### Preparation of single-stranded DNA

The procedure was carried out with modifications to our published protocol (22). The JM109 competent cells (TransGen Biotech Co., Ltd.) were transformed with the phagemids and plated onto M9 agar plates for single-colony selection. A positive single colony was then inoculated into M9 medium supplemented with ampicillin and incubated at 37°C for 12 hours with shaking. Then the bacterial culture was transferred to 2×YT media and incubated at 37°C until the OD_600_ reached 0.4–0.5. At this point, M13KO7 helper phage (NEB, N0315S) was added at a multiplicity of infection (MOI) of 1-10, and the culture was shaken at 37°C and 200–220 rpm for 30 minutes. Subsequently, kanamycin was added at the final concentration of 70 μg/mL, and the culture was continued at 37°C for an additional 3.5 hours. The culture was then centrifuged at 4°C, 4,000 rpm for 15 minutes to remove *E. coli* cells. The supernatant was collected, the phage particles were precipitated by PEG8000 4% (wt/vol) and NaCl 3% (wt/vol), followed by centrifugation at 4°C, 5,000 rcf for 30 minutes. The pellet was resuspended in TE buffer (10 mM Tris, pH 8.5), and an appropriate amount of RNase was added to remove bacterial RNA. Then, the mixture was centrifuged at 4°C and 16,000 rcf for 10 minutes to remove residual *E. coli* cells. The supernatant was carefully transferred to a new tube, labeled with the name and date, and either frozen at −20°C or used immediately.

For the extraction of phage DNA, double volumes of PPB2 solution (0.2 M NaOH, 1% SDS) were added to the phage storage solution and mixed by inversion. Then 1.5 volumes of PPB3 solution (3 M KAc, pH 5.5) were added, and the mixture was incubated on ice for 10 minutes. The mixture was then centrifuged at 4°C and 16,000 rcf for 30 minutes. The supernatant was carefully transferred to a new centrifuge tube, and an equal volume of pre-chilled absolute ethanol was added, vortexed to mix thoroughly, and incubated on ice for 30 minutes or overnight at −20°C. The mixture was centrifuged again at 4°C and 16,000 rcf for 30 minutes. The supernatant was discarded, and the resulting pellet was dissolved in 2 mL of Buffer I (100 mM NaCl, 50 mM HEPES, pH 7.0). The yield of phage ssDNA genome was quantified using a Nanodrop spectrophotometer, and the samples were stored at 4°C for up to 1 week.

### Generation of McccDNA

To isolate target ssDNA, the phage ssDNA genome was adjusted to 60 ng/μL in Buffer I. The sample was denatured by incubation in a water bath at 70°C for at least 30 minutes and was allowed to cool naturally to 37°C. Subsequently, an equal volume of Buffer II (100 mM NaCl, 50 mM HEPES, 20 mM MgCl_2_, 4 mM ZnCl_2_, pH 7.0) was added to the denatured ssDNA, and the mixture was incubated overnight at 37°C. After incubation, three volumes of ethanol were added to the mixture and then incubated at −80°C for 30 minutes or −20°C overnight, followed by centrifugation at 4°C and 16,000 rcf for 30 minutes. The supernatant was discarded, and the precipitate was dissolved in ddH_2_O. The target ssDNA was separated by 1.2% (wt/vol) agarose gel electrophoresis, recovered using NucleoSpin Gel and PCR Clean-up Kit (Macherey-Nagel, 740986.20), aliquoted, and stored at −20°C.

The minus- and plus-strand ssDNAs were mixed in a 1:1 molar ratio and annealed. The annealed products then underwent two-step ligation using T4 DNA ligase (NEB, M0202S). In the first step, samples were incubated at 16°C overnight and concentrated with the MinElute Gel Extraction Kit (QIAGEN, 28604). The second ligation step followed, with samples incubated at 16°C for at least 3 hours. Ligation products were then separated on a 1% (wt/vol) agarose gel, and the gel band containing McccDNA was excised, purified with the MinElute Gel Extraction Kit, dissolved in ddH₂O, and stored at −20°C.

### Viral nucleic acid extraction and detection

To detect HBV capsid DNA, cell samples were initially lysed with NP-40 lysis buffer (10 mM Tris-HCl, pH 8.0, 50 mM NaCl, 1 mM EDTA, 0.5% NP-40) by incubation on ice for 10 minutes. The lysed cells were transferred to 1.5 mL Eppendorf tubes and centrifuged at 10,000 rpm for 5 minutes at 4°C. The supernatant was collected, and MgAc_2_ and DNase (Sigma, DN25) were added to final concentrations of 10 mM and 100 μg/mL, respectively, followed by incubation at 37°C for 2 hours. The reaction was stopped by adding EDTA to a final concentration of 20 mM. PEG8000 was then added to a final concentration of 7%, and the sample was incubated overnight at 4°C. The mixture was centrifuged at 14,000 × *g* for 10 minutes at 4°C. The pellet was resuspended in PK buffer (10 mM Tris-HCl, pH 8.0, 100 mM NaCl, 1 mM EDTA, 1% SDS, 0.5 mg/mL proteinase K) and digested at 56°C for 2 hours. Τhe sample was extracted with saturated phenol/chloroform and then subjected to ethanol precipitation overnight at −20°C. The DNA pellet was dissolved in 20 μL ddH_2_O and analyzed by Southern blot.

Total RNA was extracted using TRIzol reagent (Thermo Fisher, 15596026). Northern blot for detection of HBV RNA, Hirt DNA extraction, and Southern blot analysis were performed as previously described (23, 24).

For quantification of preC/pgRNA and McccDNA levels, preC/pgRNA levels were measured by RT-qPCR and normalized to GAPDH. RNA was extracted, reverse transcribed, and analyzed with specific primers (pg-2455-F: 5′-ttccttggactcataaggtgggg-3′ and pg-2871-R: 5′-gctggtggaaagattctgccc-3′). McccDNA levels were measured from Hirt-extracted DNA and normalized to mitochondrial DNA using qPCR. The ratio of preC/pgRNA to McccDNA was calculated to assess the relative transcriptional activity per input McccDNA molecule.

### Immunofluorescence staining

Cell samples were initially fixed with 3.7% formaldehyde at room temperature for 10 minutes, followed by washing with PBS. The samples were then incubated in 50% ethanol for 5 minutes, transferred to 70% ethanol, and stored at 4°C for 2 hours. Afterward, the 70% ethanol was replaced with 50% ethanol for 5 minutes at room temperature, followed by a PBS wash. Subsequently, cells were re-fixed with 3.7% formaldehyde for 10 minutes at room temperature, washed with PBS, and blocked for 2 hours using blocking buffer (0.1% Triton X-100, 10% FBS). Following the blocking step, cells were incubated with the primary antibody overnight. The next day, cells were washed three times with PBST, each for 10 minutes. The cells were then incubated with the secondary antibody for 1 hour at room temperature, followed by additional PBST washes. Nuclei were stained with Hoechst 33342 (Thermo Fisher, CA, USA) at 1:1,000 dilution for 3 minutes, washed with PBS, and mounted with fluorescent mounting medium (DAKO, S3023). Samples were either stored at 4°C or immediately imaged.

### Chromatin immunoprecipitation assay

Chromatin immunoprecipitation (ChIP) was performed according to established protocols (25). For ChIP-qPCR analysis, primers P5 and P6 were used to amplify cccDNA fragments, while primers BCP + 1 fwd and BCP + 1 rev targeted nucleosome binding based on previous ChIP-Seq data in the HBV infection model (26). *Gapdh* and *Myod1* were included as reference genes to serve as cellular genomic DNA controls for amplification. Primer sequences are provided in Table S1.

### HBV infection of HepG2-NTCP

For viral inoculum preparation, HepDE19 cells were initially cultured in DMEM complete medium with doxycycline (Dox+). Afterward, the medium was replaced with doxycycline-free (Dox−) DMEM medium containing 3% FBS. The supernatant was collected every 3 days, with fresh medium added each time, over a total of four collections. The harvested supernatants were then filtered through a 0.45 μm membrane and concentrated to a final volume of 4-5 mL using a 100 kDa ultrafiltration tube (Millipore), with aliquots stored at −80°C. Viral copy numbers in each sample were quantified using the HBV DNA quantitative fluorescence diagnostic kit (Sansure Biotech). For viral infection, HepG2-NTCP cells were expanded and seeded into plates, followed by infection at an MOI of 300 using the mixture containing 4% PEG8000 and 2.5% DMSO. The plates were centrifuged at 1,000 × *g* for 1 hour at 35°C and then incubated. The next day, the cells were washed five times with PBS, and the medium was replaced with Dox− DMEM containing 3% FBS to continue the culture.

### Rapid amplification of 5′-end cDNA for HBV RNAs

The rapid amplification of 5′-end cDNA (5′ RACE) was performed following previously reported protocols (27). The forward primers PreC Gsp and Pg Gsp, along with the reverse primer Gsp2, were used to specifically amplify full-length HBV preC RNA and pgRNA. The DNA products were then ligated and sequenced using the pLB-T Fast Ligation Kit (TIANGEN, VT205).

### Micrococcal nuclease (MNase) sequencing

This method was adapted with slight modifications to the protocol described previously (25, 28, 29). Cell samples were digested with trypsin and washed twice with PBS. The cells were then resuspended in PBS at a concentration of 10^6^ cells/mL, with a reaction volume of 200 μL per sample. MNase lysis buffer (20 mM Tris-HCl, pH 7.5, 0.1% Triton X-100, 10 mM NaCl, and 2 mM CaCl_2_) was added, and the cells were lysed on ice for 10 minutes. MNase (NEB, M0247S) was diluted to the appropriate concentration with MNase storage buffer, and samples were incubated at 37°C for 20 minutes. The reaction was stopped by adding 6.25 μL of 0.5 M EGTA, followed by 43 μL of PBS containing 1 mM EGTA. The mixture was then treated with RNase, and the reaction was stopped by adding a buffer containing 0.15% SDS and 0.25 mg/mL proteinase K. Subsequently, the samples were extracted with saturated phenol/chloroform, and nucleic acids were precipitated with ethanol. The DNA pellet was dissolved in ddH_2_O. Nucleosomal DNA monomers were purified by gel extraction, dissolved in TE buffer, and sequenced on the Illumina NovaSeq 6000 platform. Sequencing data in FASTQ format were processed using Cutadapt (v1.9.1) to filter out sequences with a base quality score below 20. The filtered reads were aligned to the reference genome using Bowtie2 (v2.3.5.1), and alignment results were further filtered and sorted with SAMtools (v1.7). Nucleosome distribution was analyzed using DANPOS3 software, and the results were visualized with IGV (30). The nucleosome level was calculated as the ratio of reads within the interest region (span = 10) to the average reads across the whole genome, and the curve fitting was generated by GraphPad’s fit spline feature.

### Statistical analysis

Data were analyzed using GraphPad Prism 8.0 (GraphPad Software Inc.). Statistical analyses were performed using the Student’s t test, and data were presented as the mean ± standard deviation (SD). A *P* value < 0.05 was considered to be statistically significant. Statistically significant differences are indicated by * for *P* < 0.05, ** for *P* < 0.01, and *** for *P* < 0.001.

## RESULTS

### Construction of HBV cccDNA from M13 phage-derived ssDNA

**Fig 1:**
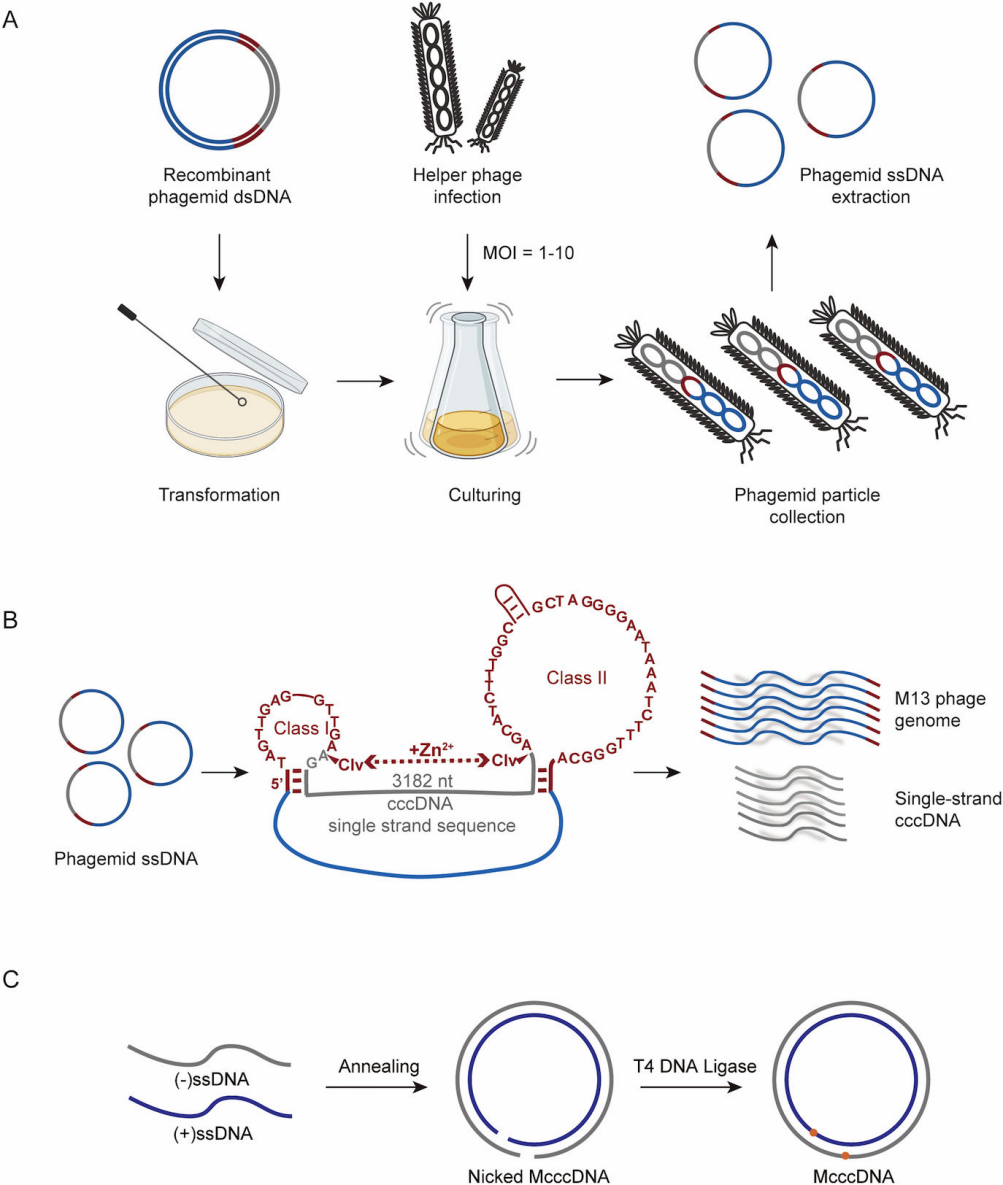
Schematic diagram of McccDNA production utilizing the M13 phage ssDNA system. (**A**) The construction of the recombinant phagemid is illustrated. The HBV cccDNA sequence (shown in gray), which was flanked by class I and class II deoxyribozymes (indicated in red), was inserted into the phagemid backbone (indicated in blue). The constructed phagemids were transformed into JM109-competent cells and amplified until the OD_600_ of the culture reached about 0.4–0.5. The cells were then infected with helper phage at an MOI of 1–10 and further cultured for 4 hours. The phage particles were collected, followed by lysis and recovery of the recombinant phagemid ssDNA. (**B**) The schematic diagram details the M13 phage-based ssDNA production system. The zinc ions present in Buffer II facilitate the cleavage at specific sites of Class I and Class II deoxyribozymes, enabling the separation of the M13 phage genome backbone and the single-stranded McccDNA. (**C**) The schematic diagram illustrates the cyclization and ligation of ssDNA to form McccDNA.

Building on the *in vitro* formation of recombinant relaxed circular DNA (RrcDNA) through ssDNA annealing and ligation, we aimed to investigate the methods for constructing HBV cccDNA devoid of exogenous sequences using ssDNA (7, 31). Initially, we constructed two phagemids, one containing the positive-strand and the other containing the negative-strand sequences of HBV cccDNA, respectively, flanked by Class I and Class II deoxyribozyme sequences. These deoxyribozymes are capable of cleaving phosphodiester bonds at specific sites in the presence of zinc ions. Following the transduction of transformed *E. coli* with M13 helper phage, we lysed the progeny recombinant M13 phage particles and extracted their genome (Fig. 1A). Subsequently, under the action of the deoxyribozymes, this process separates the phage genome backbone from the target HBV ssDNA sequence (Fig. 1B). Annealing the full-length HBV ssDNA derived from M13 phage results in the formation of a double-stranded circular DNA containing only two nicks (Nicked McccDNA). Subsequently, the addition of T4 DNA ligase repairs these nicks, yielding covalently closed circular HBV cccDNA (McccDNA) (Fig. 1C).

**Fig 2:**
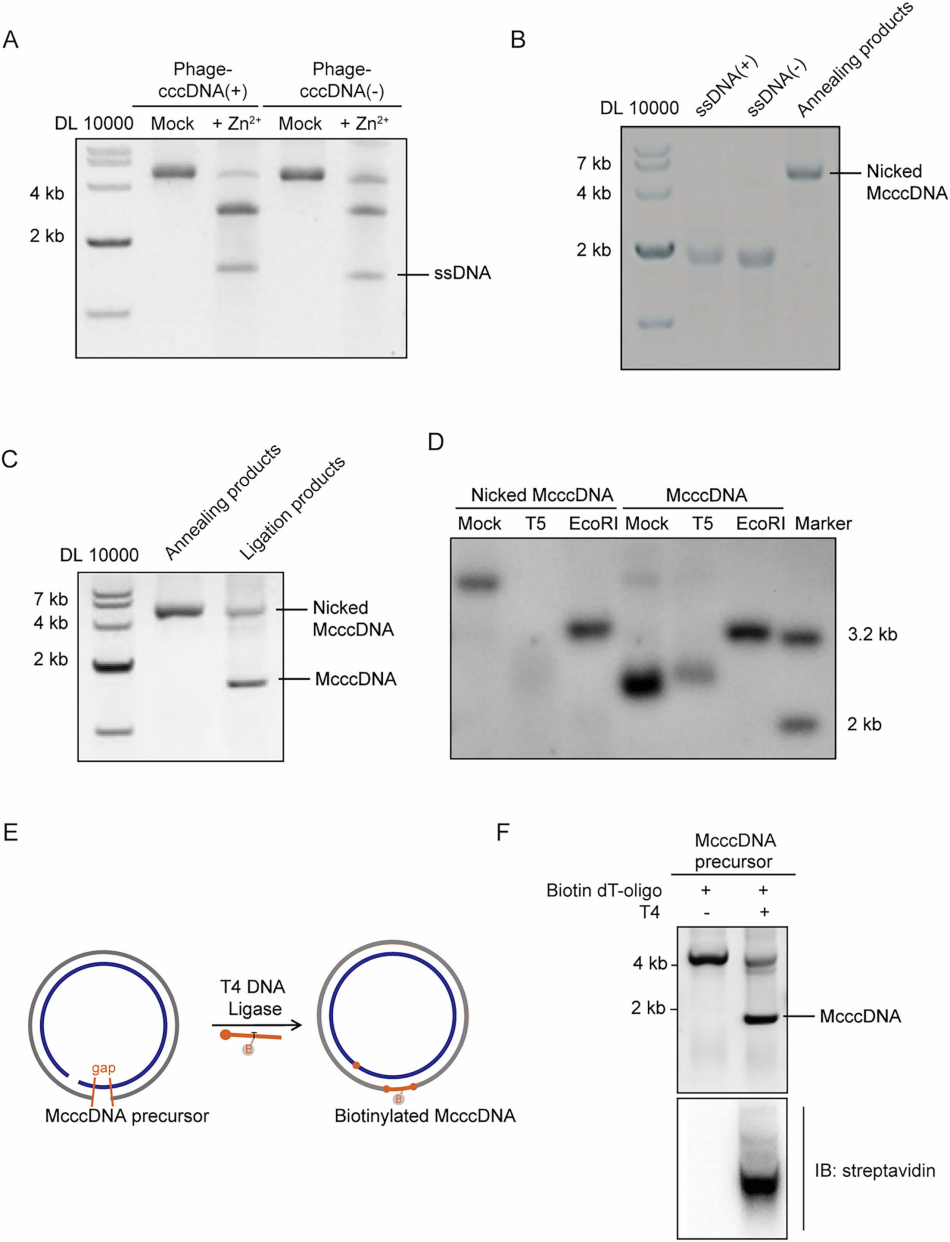
Generation of *in vitro*-cyclized McccDNA model based on M13 phage-single-stranded DNA production system. (**A**) The generation of single-stranded HBV cccDNA via deoxyribozyme-mediated cleavage was confirmed through urea-agarose gel electrophoresis. (**B**) The purified positive-strand, negative-strand ssDNA, and annealing products were monitored by agarose gel (0.7%) electrophoresis. (**C**) The annealing products were treated with T4 DNA ligase. The electrophoretic mobilities of annealing and ligation products were analyzed and compared on agarose gel. (**D**) Verification of the covalently closed circular form of McccDNA through digestion with T5 exonuclease or the restriction enzyme EcoRI, followed by detection using Southern blot analysis. (**E**) Schematic diagram of the generation of biotinylated McccDNA using truncated ssDNA. (**F**) The annealing products of truncated negative-strand ssDNA were ligated with biotin dT-oligo, followed by agarose gel electrophoresis. DNA was also transferred to nylon membrane and immunoblotted with streptavidin-horseradish peroxidase conjugate. Abbreviations: T4, T4 DNA ligase; T5, T5 exonuclease; IB, immunoblotting.

The recombinant phage genome was extracted and incubated overnight in a zinc ion-containing buffer, resulting in successful separation of HBV ssDNA (Fig. 2A). The positive- and negative-strand ssDNA were purified from the gel and annealed at equal masses, followed by the addition of T4 DNA ligase for ligation. The results demonstrated that the purified ssDNAs were effectively annealed into circular double-stranded DNA (Fig. 2B). The ligation product exhibited a distinct new band at the 2.0 kb position, which corresponds to the migration rate of supercoiled HBV cccDNA (Fig. 2C). Further verification through Southern blot analysis confirmed the relative resistance of the purified ligation products to T5 exonuclease, and after treatment with EcoRI restriction endonuclease, the products migrated to the 3.2 kb position, corroborating their covalently closed circular structure (Fig. 2D). Moreover, producing the truncated ssDNA from M13 phage genome, subsequently annealed and ligated with biotinylated oligonucleotides, represents a promising methodology for the synthesis of biotinylated McccDNA (Fig. 2E and F). Collectively, these results indicate that HBV ssDNA derived from M13 phage can undergo annealing and ligation to form HBV cccDNA *in vitro*.

### Characterization of McccDNA model

**Fig 3:**
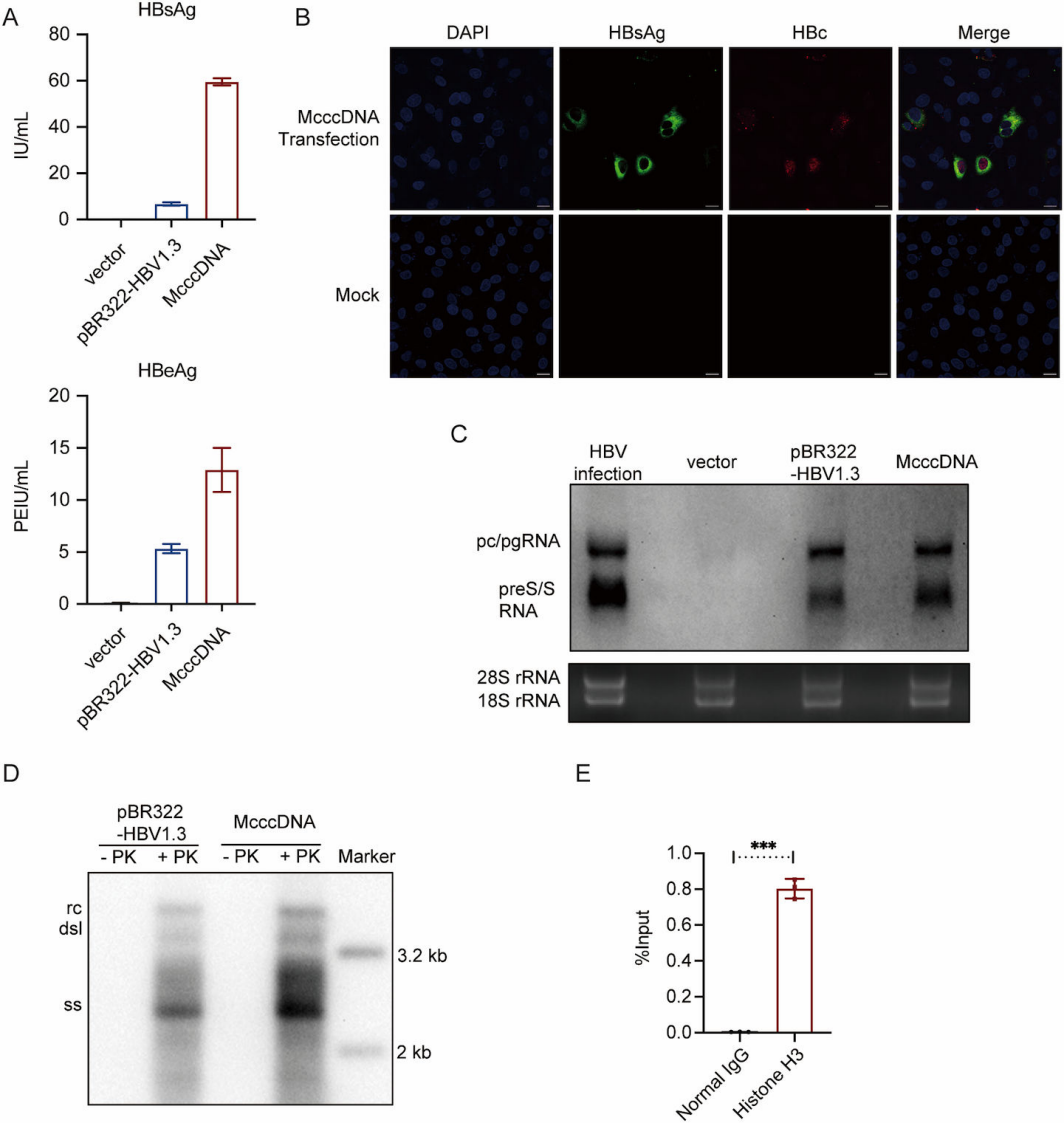
*In vitro*-cyclized McccDNA model supports high-level HBV replication in transfected cells driven by minichromosomes. HepG2-NTCP cells were transfected with 100 ng of either McccDNA or pBR322-HBV1.3 in 24-well plates. After 72 hours, the supernatant was collected and analyzed for (**A**) HBsAg and HBeAg levels using ELISA. (**B**) Immunofluorescence staining of fixed cells was performed to detect HBsAg (green) and HBc (red). (**C**) Total RNA was extracted, and HBV RNA was detected via Northern blotting. HepG2-NTCP cells infected with HBV at MOI 300 for 7 days; 10 μg of extracted RNA was used for Northern blot as a positive control. (**D**) After 5 days of DNA transfection, cytoplasmic DNA was isolated, and HBV capsid DNA was analyzed by Southern blotting. (**E**) ChIP was performed on HepG2-NTCP cells transfected with McccDNA after 2 days, using antibodies to Histone H3 or control IgG. Data are plotted as the percentage of input. ***P < 0.001.

By transfecting HepG2-NTCP cells, we conducted a comprehensive assessment of the transcription, replication, and antigen expression capabilities of McccDNA. Similar to pBR322-HBV1.3, which contains the standard 1.3-unit HBV genome and was included as a positive control, transfection with McccDNA also supported the expression of key viral proteins, including HBsAg, HBeAg, and HBc (Fig. 3A and B). We also performed a time-course McccDNA transfection experiment and collected samples within 7 days, which indicated that McccDNA could sustain antigen secretion and HBV replication (Fig. S1A). Additionally, McccDNA efficiently transcribed the major HBV RNAs and replicated to package viral particles (Fig. 3C and D). The supernatant from the transfected cells was collected, concentrated via ultrafiltration, and then incubated with newly seeded HepG2-NTCP cells for 7 days. Subsequent analyses demonstrated detectable levels of HBV antigens in the supernatant (Fig. S1B and C), while immunofluorescence assays confirmed the presence of HBsAg in the cytoplasm (Fig. S1D). These findings indicate that the McccDNA transfection system successfully produced mature HBV particles with infectivity. Furthermore, a ChIP assay was performed to confirm the formation of minichromosomes from McccDNA in the nucleus, revealing that histones were associated with McccDNA (Fig. 3E). In summary, the McccDNA transfection system can form cccDNA minichromosomes in the nucleus and effectively support active transcription, replication, and viral packaging processes.

### Efficacy of anti-HBV therapies in McccDNA model

**Fig 4:**
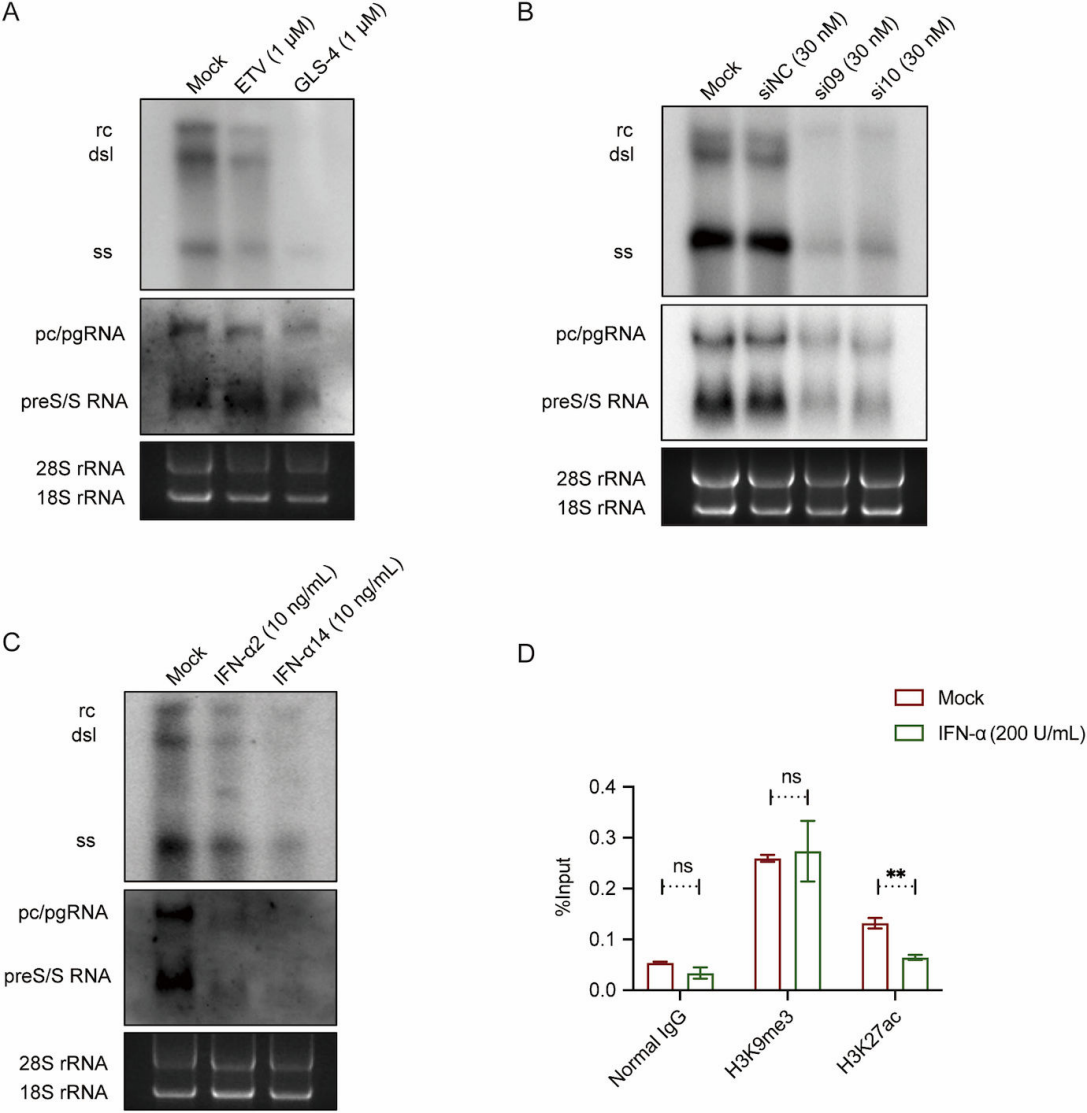
*In vitro*-cyclized McccDNA model actively responds to anti-HBV drugs. HepG2-NTCP cells were transfected with 100 ng of McccDNA. After 6 hours, the medium was changed, and cells were subsequently treated with (**A**) small molecule inhibitors (ETV, GLS-4) or (**C**) interferons (IFN-α2, IFN-α14) at the indicated concentrations. The medium was refreshed every 3 days. (**B**) After 24 hours of DNA transfection, siRNA was transfected into cells at the indicated dosages. Total RNA was extracted at day 3, and HBV RNA levels were analyzed through Northern blot. Cytoplasmic DNA was harvested on day 5, and HBV capsid DNA was detected through Southern blot. (**D**) HepG2-NTCP cells were transfected with 500 ng of McccDNA in 10 cm culture dishes, followed by treatment with universal IFN-α after 24 hours. Cell samples were collected 48 hours post-transfection, and the epigenetic modifications of transfected McccDNA were assessed using ChIP assays. Antibodies against H3K9me3, H3K27ac, or control IgG were used. Data are plotted as the percentage of input. **P < 0.01; ns, not significant (*P* ≥ 0.05).

We further investigated the efficacy of anti-HBV drugs targeting various stages of the HBV lifecycle within the McccDNA model. In McccDNA-transfected cells, the nucleoside analog entecavir (ETV) and the capsid assembly modulator GLS-4 demonstrated significant inhibitory effects on viral replication, resulting in a notable reduction in HBV DNA levels, while having no impact on HBV RNA levels (Fig. 4A). To explore the potential of oligonucleotide therapy targeting HBV RNA degradation, we synthesized two small interfering RNAs (siRNAs) targeting the open-reading frame of the HBV X protein and transfected them into cells containing McccDNA. The results indicated that both total HBV RNA and HBV DNA levels were significantly decreased (Fig. 4B). Additionally, treatment of the transfected cells with IFN-α, which inhibits HBV replication and promotes viral RNA degradation, led to decreases in both HBV RNA and DNA levels. Among the two IFN-α subtypes evaluated, the IFN-α14-treated group exhibited a more pronounced decrease, consistent with our previous observations (32) (Fig. 4C). Furthermore, we also assessed changes in epigenetic markers following IFN-α treatment. The active transcription marker acetylated H3K27 (H3K27ac) was significantly decreased, while the repressive transcription marker histone 3 lysine 9 trimethylation (H3K9me3) remained at a similar level (Fig. 4D). All these results indicate that the McccDNA model serves as a valuable platform for investigating the efficacy of anti-HBV drugs, as well as elucidating the underlying mechanisms of viral gene regulation (33, 34) and replication (20, 32).

### Impact of exogenous sequences on HBV cccDNA activity

**Fig 5:**
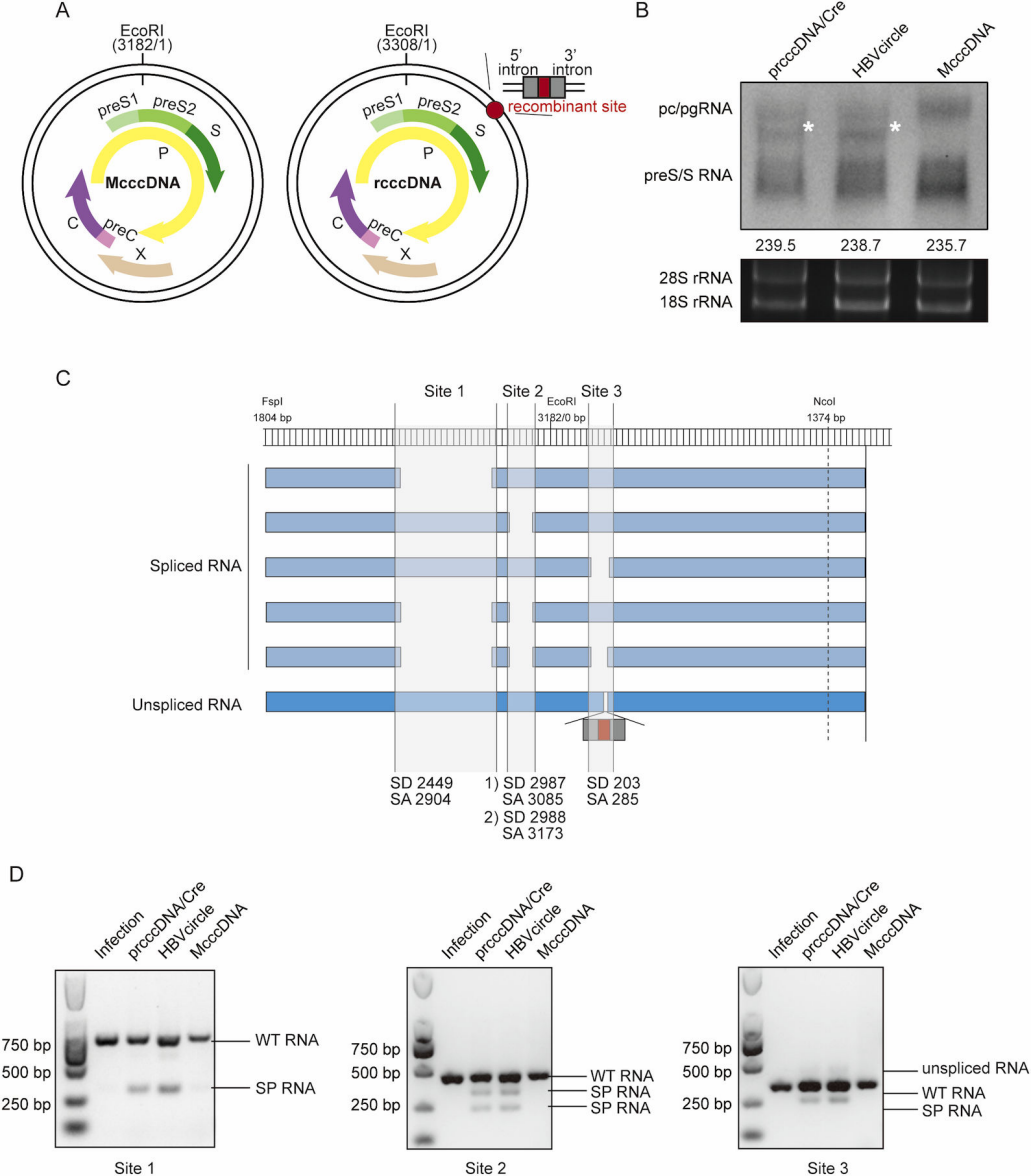
The presence of exogenous sequences affects the precise transcriptional process of HBV cccDNA. (**A**) In contrast to the McccDNA model, the rcccDNA model incorporates exogenous sequences, specifically recombinase recognition sites that are flanked by introns. The splicing process mediated by the inserted intron after transcription removes the recombination sites, thereby ensuring that no exogenous sequences are present in the HBV RNA. The position of these insertions (205–330 bp) is depicted above. (**B**) HepG2-NTCP cells were transfected with 100 ng of either McccDNA or rcccDNA. Total RNA was extracted on day 3, and HBV RNA levels were analyzed using Northern blot. (**C**) The analysis of preC/pg RNA-specific 5′ RACE amplicons in rcccDNA-transfected cells compared to those from the infection system. The primary splicing sites of these amplicons are indicated as Site 1 (SD 2449, SA 2904), Site 2 (SD 2987, SA 3085; SD 2988, SA 3173), and Site 3 (SD 203, SA 285). SD represents the splice donor, and SA represents the splice acceptor. Nucleotide numbering is based on authentic cccDNA. The recombinant sequence contained in the unspliced RNA refers to 5′-GTAAGTATCAAGGTTACAAGACAGGTTTAAGGAGACCAATACCCCAACTGGGGTAACCTTTGGGCTCCCCGGGCGCGGCACCTATTGGTCTTACTGACATCCACTTTGCCTTTCTCTCCACAGG-3′ (**D**) Agarose gel electrophoresis of cDNA amplicons was performed using primers flanking the spliced sites. The main band corresponds to wild-type HBV RNA transcripts (WT RNA) in the infection system. The truncated band represents spliced HBV RNA transcripts (SP RNA) in the rcccDNA system, while the extended band corresponds to unspliced HBV RNA transcripts containing introns and recombinant sites in the rcccDNA system.

HBV cccDNA sequence is highly compact, with overlapping ORFs, indicating that a single point mutation can affect the expression and function of multiple viral proteins. Therefore, the McccDNA model, which lacks exogenous sequences, provides a more accurate representation of natural cccDNA. To investigate the impact of exogenous sequences on HBV cccDNA activity, we compared the McccDNA model with recombinant cccDNA (rcccDNA) containing exogenous sequences (Fig. 5A). Following the transfection of equal amounts of rcccDNA and McccDNA, we observed significantly lower levels of antigen expression in the rcccDNA system (Fig. S2A). The prokaryotic sequence backbone of prcccDNA, resulting in a molecular weight distinct from that of HBVcircle and McccDNA, complicates normalization efforts. Consequently, we compared HBV replication between the HBVcircle and McccDNA transfection groups, observing a weaker HBV capsid DNA signal in cells transfected with HBVcircle by Southern blot (Fig. S2B), while the levels of transfected HBV genomes were comparable between the groups (Fig. S2C). However, Northern blot analysis showed no significant difference in overall HBV RNA transcription levels between the two systems, though a distinct band, migrating more slowly than the 3.5 kb transcripts, was detected in the rcccDNA system (Fig. 5B).

To explore whether this additional band is associated with the functional activity of the rcccDNA molecule, we employed 5′ RACE to amplify HBV transcripts in the transfection groups, using the infection group as a control (Fig. S3A). To enrich for transcripts related to the 3.5 kb RNA, we treated cells with the capsid assembly modulator JNJ-632 to accumulate pgRNA for cloning. This treatment did not affect cell viability (Fig. S3B). Using primers specific to pg- or preC RNA in the full-length 5′ RACE cDNAs, we confirmed the enrichment (Fig. S3C and D). Sequencing of the major bands revealed splicing events within the 3.5 kb transcripts in the rcccDNA system, with splice sites concentrated at three sites, which were distinct from the insertion sites of the exogenous sequences in rcccDNA (Fig. 5C). PCR amplification using primers flanking these splice sites confirmed the presence of truncated RNA transcripts in the rcccDNA system (Fig. 5D), and the relative abundance of these truncated RNAs was quantified through analysis of the quantitative RT-PCR results (Fig. S2D). These results suggest that the introduction of exogenous sequences in rcccDNA increases the possibility of HBV RNA splicing, leading to the production of truncated pgRNA and preC RNA. Specifically, the truncated pgRNA results in premature termination of HBV polymerase translation, impairing the reverse transcription of pgRNA, while the truncated preC/pgRNA causes a frameshift mutation, leading to defective HBeAg and HBc expression. In contrast, the McccDNA model without exogenous sequences is a more accurate system for studying HBV cccDNA and its biological activity.

### MNase-seq mapping of nucleosome binding on HBV cccDNA minichromosome

**Fig 6:**
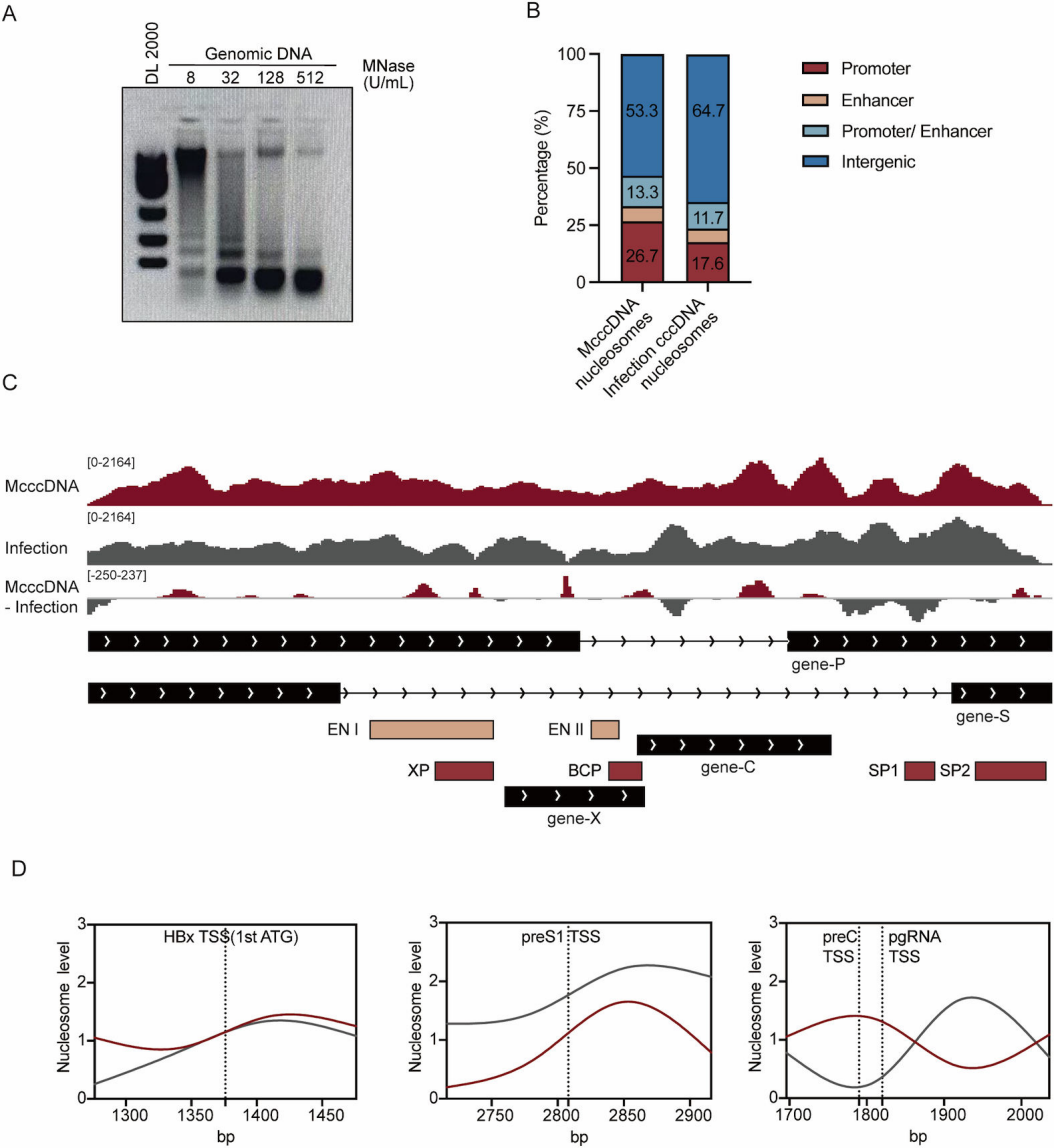
Mapping of nucleosome binding on HBV cccDNA minichromosomes by MNase-seq. (**A**) The DNA products of digestion with a gradient of MNase concentrations were subjected to gel electrophoresis. Mononucleosome-sized DNA purified from 128 U/mL of the MNase treatment group was analyzed by high-throughput sequencing, and the reads were aligned to the HBV genome (subtype ayw; GenBank accession no. V01460.1), as well as Mono-seq from the infection system (accession no. GSM1670898). (**B**) The percentages of nucleosome-binding sites were quantified across different genomic regions, including promoters, enhancers, promoter-enhancer overlapping regions, and all remaining regions, which were categorized as intergenic. The nucleosome-binding sites were identified using DNAPOS. (**C**) Nucleosome differential signals between these two systems are plotted as red (positive) or gray (negative) areas at the bottom track. (**D**) Analysis of nucleosome levels around TSSs of HBx, preS1, and preC/pg RNA was conducted. The red line denotes data from the McccDNA-transfected group, while the gray line shows the results from the infected group as indicated. The nucleosome level was calculated as the ratio of reads within the interest region (span = 10) to the average reads across the whole genome. Abbreviations: EN, enhancer; XP, HBx promoter; BCP, basic core promoter; SP, surface promoter.

The nucleosome arrangement of the HBV minichromosome has remained unclear, largely due to the limited sequencing reads from infection systems. To address this, we aimed to explore the nucleosome characteristic of McccDNA and their similarity to the natural minichromosome, and to identify specific nucleosome-binding sites. We employed the MNase-seq method to detect binding sites identified as DNA sequences protected from MNase digestion. Upon treatment with gradient concentrations of MNase, all the cell nucleus DNA exhibited DNA ladder-like patterns (Fig. 6A). The mononucleosome-sized DNA was subsequently purified from the gel and subjected to sequencing. The data were filtered, aligned to the HBV genome, and normalized to Mono-seq (26), which was generated from infected samples following probe enrichment. Statistical analysis of the sequencing data indicated a high degree of similarity between the DNA signals from the McccDNA system and the infection system. Nucleosomes are predominantly enriched in the intergenic region of the HBV genome, with a pronounced difference in enrichment observed in the promoter region and only minimal variation in the enhancer region (Fig. 6B). Additionally, nucleosome peaks were found to be positioned nearly identically in both systems, exhibiting a 72.97% similarity (Fig. 6C). Given that the positioning of nucleosomes near promoters can impact the transcriptional activity of genes, we conducted a comparative analysis of nucleosome distribution around transcriptional start sites (TSS) (Fig. 6D). The results revealed consistent nucleosome localization patterns around the TSS regions of HBx and preS1 in both systems, particularly downstream of the TSS, indicating a higher accessibility of the promoter region. However, distinct nucleosome occupancy around the preS2 region was not observed in either system. Furthermore, the nucleosome positioning around the preC/pg RNA region suggests a more open state in the infection system, potentially reflecting the more active transcriptional state of cccDNA in the HBV infection process.

Despite minor differences that may arise from sequence bias during the probe enrichment process of Mono-seq, these observations highlight the potential of the McccDNA model as a valuable tool for studying the dynamic organization of nucleosomes on natural cccDNA minichromosomes.

### Characterization of the key viral protein-deficient cccDNA by generated McccDNA model

**Fig 7:**
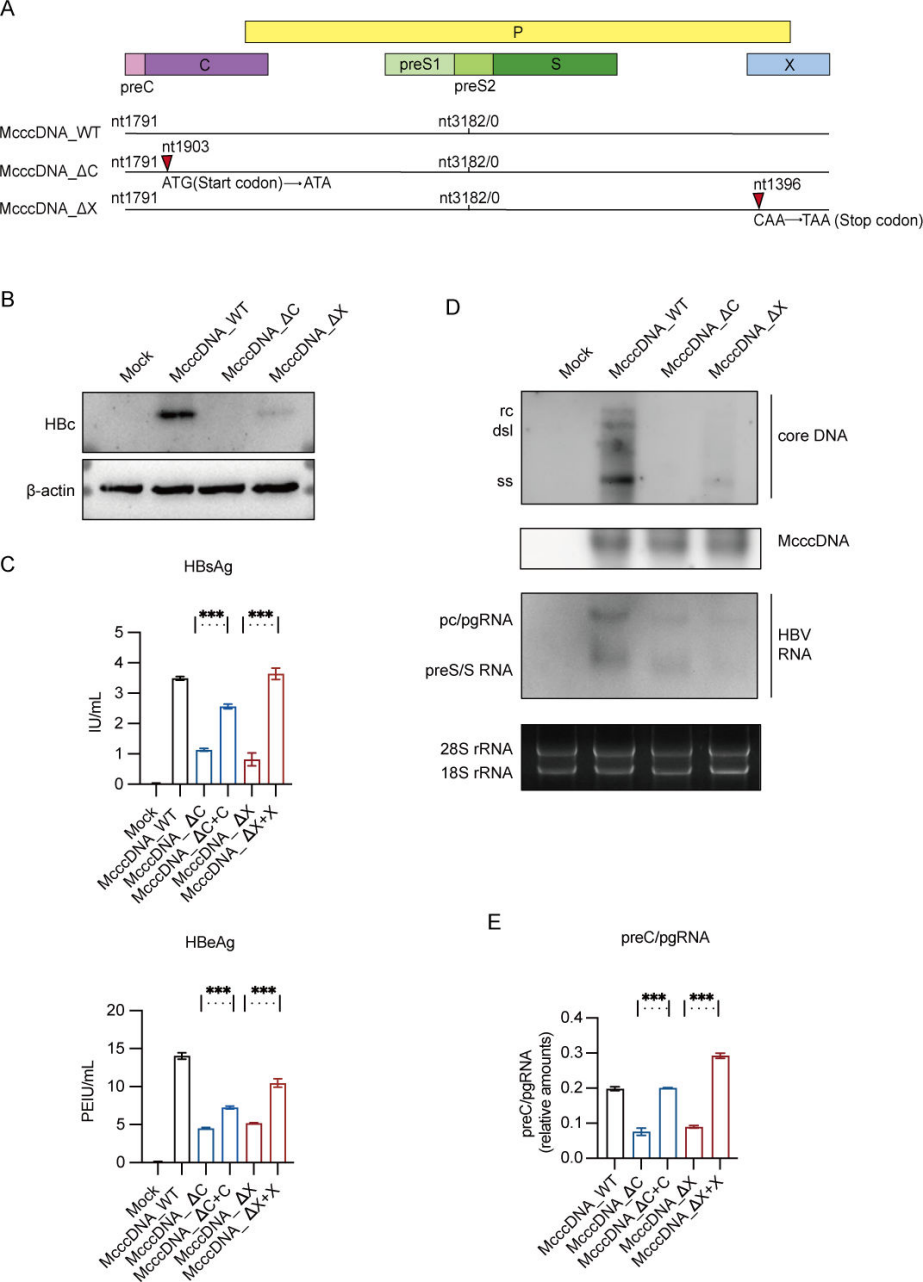
Construction and characterization of viral protein-deficient McccDNA. (**A**) Schematic representation of WT and viral protein-deficient construct. The start codon of the core gene was mutated to ATA in McccDNA_ΔC, thereby abolishing HBc translation. A premature stop codon was introduced at nt1396 within the X gene in McccDNA_ΔX, leading to loss of HBx expression. Mutation sites are indicated by red inverted triangles. (**B**) HepG2-NTCP cells were transfected with the same amount of McccDNA, McccDNA_ΔC, or McccDNA_ΔX. The pcDNA3.1-3xFlag-HBx and pTruf-HBc were co-transfected with McccDNA, while the control group was transfected with the same amount of the pcDNA3.1 vector. HBc expression was detected by Western blot. (**C**) The supernatants were collected on day 3, and HBsAg or HBeAg levels were analyzed using ELISA. (**D**) Core DNA and Hirt DNA were extracted on day 5 and detected by Southern blot. (**E**) HBV RNA was extracted on day 3 and detected by Northern blot and RT-qPCR. The relative amount of preC/pgRNA was normalized to the cccDNA amount in Hirt DNA extraction. ***P < 0.001.

It has been reported that both HBc and HBx proteins can bind to cccDNA minichromosomes, which, in turn, may contribute to the transcriptional activity of cccDNA (10, 35–39). However, due to the inherently low efficiency of cccDNA formation during HBV infection, elucidating the regulatory mechanisms of these viral proteins on cccDNA minichromosomes remains challenging. To address this issue, we constructed the HBc-deficient (McccDNA_ΔC) and HBx-deficient (McccDNA_ΔX) McccDNA model through targeted single-site mutation in the viral genome (Fig. 7A). As expected, McccDNA_ΔC and McccDNA_ΔX resulted in reduced antigen secretion when compared to the wild-type McccDNA (McccDNA_WT), and restoration of the deleted viral protein in each group led to a marked recovery of antigen expression (Fig. 7B and C). Following transfection with the same amounts, both mutants exhibited significant reductions in HBV RNA and DNA levels compared to the McccDNA_WT, indicating suppressed transcriptional activity and viral replication (Fig. 7D). The results were further confirmed by quantitative analysis of HBV pgRNA normalized to McccDNA copy number at 72 hours post-transfection (Fig. 7E). Collectively, these results demonstrate that McccDNA is a robust platform for manipulating the HBV genome and facilitates detailed virological studies.

## DISCUSSION

HBV cccDNA serves as the template for HBV transcription and replication within the nuclei of infected hepatocytes, establishing a persistent cccDNA reservoir that poses a critical challenge to curing chronic hepatitis B. In terms of structure, cccDNA binds with histones to form a minichromosome, adopting a stable, chromatin-like conformation. At the sequence level, cccDNA contains four promoters and two enhancers with binding sites for liver-specific transcription factors and nuclear receptors, which promote active transcription within hepatocyte nuclei (33, 34, 40). Recently, researchers have utilized an *in vitro* method to remodel cccDNA mini-chromosomes and discovered that chromatinization of cccDNA promotes transcription of the critical HBV X gene, emphasizing the importance of both sequence and structure in determining the activity of cccDNA (41). Given these complexities, developing a cccDNA research model that accurately reflects the natural characteristics of cccDNA is crucial for advancing our understanding of its unique molecular architecture and biological function.

In natural infection models or in stably transfected cell lines, this low copy number necessitates highly sensitive detection methods, and the abundant HBV DNA in these systems often introduces substantial background signal, further complicating precise analysis. To address these limitations, the rcccDNA model employs recombinases to circularize the linear HBV genome containing recombination sites, generating rcccDNA with substantially increased cccDNA copy numbers in the nucleus through transfection. During recombination, the plasmid backbone is removed, producing a molecule comparable in size to native cccDNA. This model simulates cccDNA minichromosomes and supports applications in studies of epigenetic state transition (25), host factor identification (42, 43), and antiviral drug evaluation (17, 44–46). However, the rcccDNA model retains recombination site insertions, which impacts replication functionality and antigen expression, potentially limiting its fidelity in fully mimicking native cccDNA behavior.

Here, we developed a novel *in vitro* synthesis method to produce full-length cccDNA using M13 bacteriophage single-stranded DNA preparation technology. Leveraging the rapid replication of M13 bacteriophage, this approach yields approximately 1 mg of single-stranded DNA from a three-liter bacterial culture. Although the multi-step purification process introduces some losses, the final yield of cccDNA reaches approximately 10%, providing sufficient microgram-level quantities for *in vitro* experiments while avoiding the introduction of DNA methylation. The generated McccDNA can successfully assemble into minichromosomes within the nucleus, undergo epigenetic regulation, exhibit active replication and antigen expression, and demonstrate effective responsiveness to multiple antiviral drugs. MNase-seq analysis further validated that the histone arrangement within minichromosomes formed by McccDNA transfection closely resembles that of cccDNA from natural HBV infection, identifying multiple specific nucleosome-binding sites. Additionally, McccDNA offers an enhanced and manipulatable platform for the analysis of chromatin structure, enabling the generation of specifically labeled cccDNA molecules without the necessity of HBV DNA fragment enrichment via probes. The recurrence of the cccDNA chromatin architecture supports the suitability of McccDNA as a model for studying the structural features and regulatory mechanisms.

The elucidation of the constitution of the cccDNA minichromosomes-associated DNA-protein complex remains a critical endeavor in advancing the identification of novel restriction factors for the development of anti-HBV drugs. Although the nucleosome-binding sequence on cccDNA has been identified through *in vitro* reconstructed cccDNA models and our results, the binding pattern may vary in infection condition, potentially leading to changes in interactions with host factors. Therefore, the context-specific interactions between cccDNA and host factors warrant further investigation. Two distinct strategies have been proposed for the screening of binding host factors. The first approach involves the incubation of cell lysate with rcccDNA models *in vitro* (42, 43), while the second method entails the baiting of cccDNA molecules within the cellular nucleus (38, 47), followed by mass spectrometry detection. Based on our MNase-seq analysis, the latter strategy appears to be more adept at capturing the binding proteins due to the presence of histone, transcription factors, and the RNA pol II complex arranged in specific sites on cccDNA (48). Conversely, the *in vitro* incubation option may be more effective in enriching sequence-specific binding proteins, which could potentially be diminished within the intranuclear state. The biotinylation of McccDNA confers a distinct advantage, rendering it a superior model for screening purposes.

Our study further revealed that the insertion of exogenous sequences into cccDNA results in decreased replication activity and reduced antigen expression. These changes are associated with the generation of various splice variants of preC RNA and pgRNA at specific sites, which differ from the known splicing patterns observed in clinical settings (49–51). Therefore, the different viral protein expression patterns caused by RNA splicing may introduce difficulties in directly comparing results from the rcccDNA system to those from other models (39). Due to the absence of distinct preS/S-related bands with lower migration rates in Northern blot analysis, we did not conduct full-length sequencing of these transcripts, leaving the potential impact of exogenous sequences on S protein expression to be further investigated.

Although McccDNA shares sequence similarity with natural HBV cccDNA and successfully forms the minichromosome within the nucleus, this experimental approach has notable limitations. McccDNA can only be introduced into human hepatocyte cell lines via transfection, while facilitating controlled comparative analyses of cccDNA chromatin organization, differences in cccDNA copy number relative to authentic infection should be considered when interpreting population-level chromatin data. As cell lines continue to divide, transfected McccDNA gradually diminishes, reflecting the loss of cccDNA during mitosis (52, 53). While the McccDNA system differs significantly from natural infection models in terms of initial cccDNA copy number, it offers a unique opportunity to study cccDNA loss dynamics during cell division. Moreover, by combining this model with advanced techniques such as mass spectrometry, it could be further utilized to identify factors involved in cccDNA stability maintenance and clearance, providing new insights into the regulation of cccDNA persistence.

In conclusion, this study establishes a novel system for investigating HBV cccDNA minichromosomes, effectively addressing key limitations inherent in existing cccDNA research models. This system offers a suitable and efficient platform for in-depth exploration of cccDNA minichromosome structure and function, supporting the development and assessment of new therapeutic strategies targeting cccDNA.

## Data Availability

The MNase-seq data sets involved in the study are available in GEO at the NCBI under the accession number GSE291625.

## SUPPLEMENTAL MATERIAL

Supplemental material jvi.00035-26-s0001.pdf Fig. S1 to S3; Tables S1 to S3.

ASM does not own the copyrights to Supplemental Material that may be linked to, or accessed through, an article. The authors have granted ASM a non-exclusive, world-wide license to publish the Supplemental Material files. Please contact the corresponding author directly for reuse.
