# Supplementary material for: Generation of HBV cccDNA using single-stranded M13 phage DNA for authentic minichromosome functionality
Source: J Virol. 2026 May 14;100(6):e00035-26. doi: 10.1128/jvi.00035-26 (PMC13288475; doi:10.1128/jvi.00035-26)
Supplement: Supplemental material — Fig. S1 to S3; Tables S1 to S3. [file jvi.00035-26-s0001.pdf]

Supplementary Materials for

Generation of HBV cccDNA Using Single-Stranded M13 Phage DNA for Authentic Minichromosome Functionality

Yumeng Li [#], Ting Hua [#], Menghan Hao, Liman Chen, Wenjun Huang, Zhong Fang, Asha Ashuo, Yaming Li, Zhigang Yi, Hongzhou Gu[*], Zhenghong Yuan [*], Jieliang Chen [*]

Corresponding authors:

hongzhou.gu@fudan.edu.cn, zhyuan@shmu.edu.cn, jieliangchen@fudan.edu.cn

Figure S1 HBV particles generated from McccDNA-transfected cells are infectious to HepG2-NTCP cells.
Figure S2 The McccDNA model possesses higher antigen expression and replication ability compared to rcccDNA models.
Figure S3 Amplification of major HBV transcripts in different systems by 5'RACE.
Table S1 Primer sequences used for this study.
Table S2 Antibodies used in this study.
Table S3 Sanger sequencing for McccDNA.

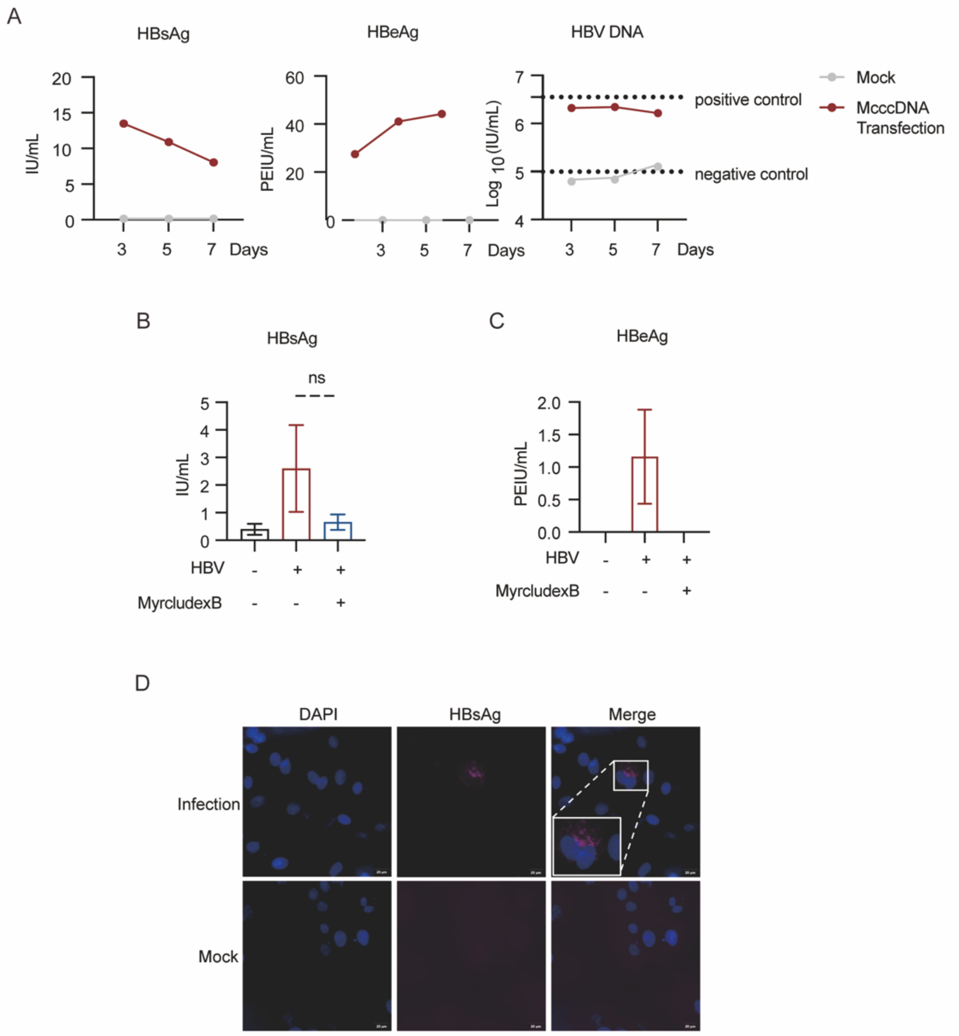

Figure S1 HBV particles generated from McccDNA-transfected cells are infectious to HepG2-NTCP cells.

(A) Time-course analysis of antigen expression and HBV DNA levels in the McccDNA transfected HepG2-NTCP. Viral antigens and HBV DNA were measured at days 3, 5, and 7 post-transfection. (B-C) McccDNA was transfected into HepG2-NTCP cells, and the supernatant was collected on days 3, 5 and 7. The supernatant containing HBV particles were then concentrated to infect HepG2-NTCP cells for 7 days. HBsAg (B), HBeAg (C) levels in the cell supernatant with or without MyrcludexB treatment were measured by ELISA, and intracellular HBsAg (D) expression was detected by immunofluorescence staining.

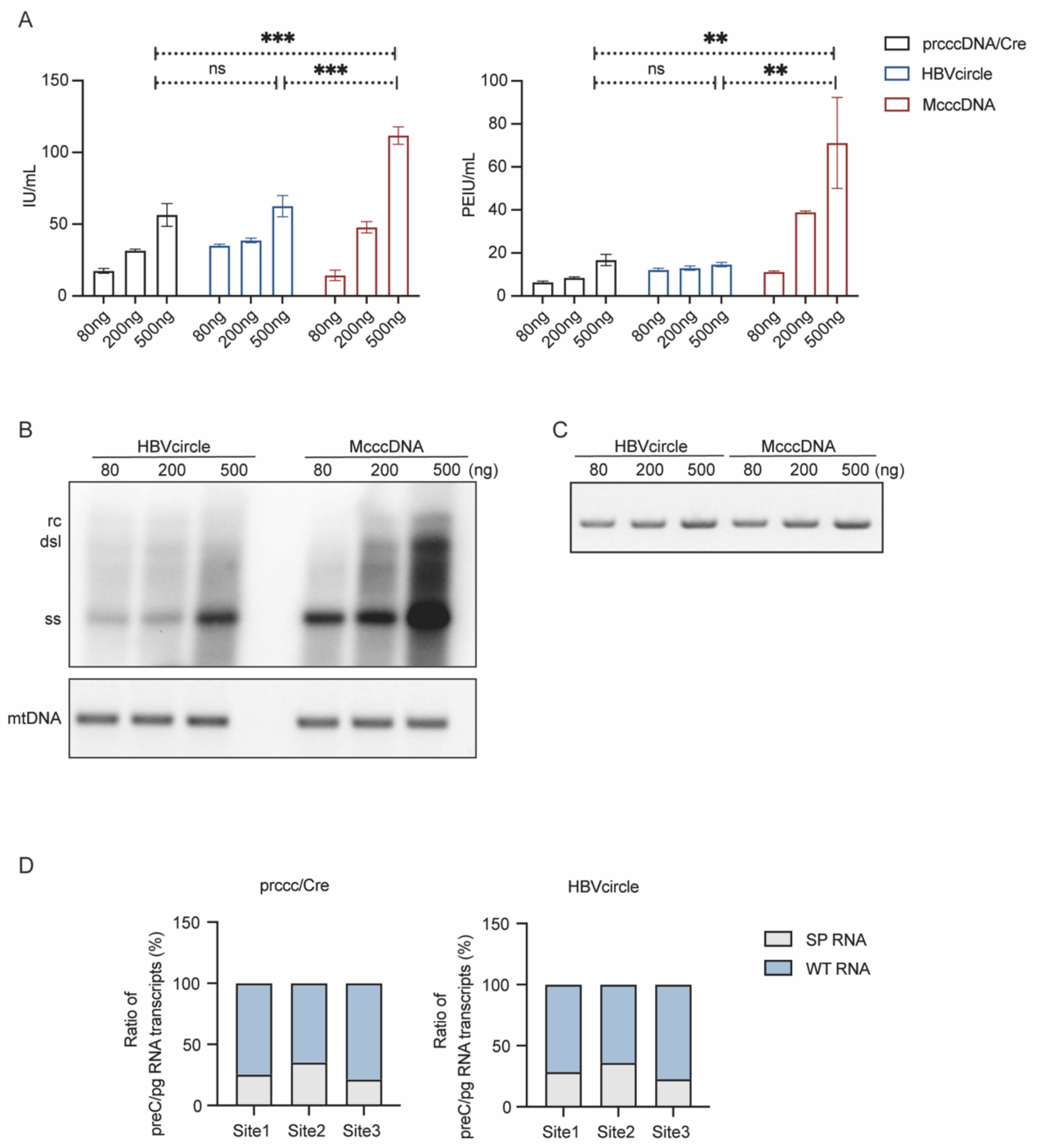

Figure S2 The McccDNA model possesses higher antigen expression and replication ability compared to rcccDNA models.

HepG2-NTCP cells were transfected with equal quality of McccDNA or rcccDNA as indicated. 5 days after transfection, (A) HBsAg and HBeAg in cell supernatants were measured by ELISA, (B) capsid DNA was extracted and detected by Southern blotting. (C) Agarose gel electrophoresis of PCR products amplified with HBV-specific primers using Hirt DNA as templates. (D) Agarose gel electrophoresis of cDNA amplicons was performed using primers flanking the spliced sites and the relative ratios of the truncated HBV preC/pg RNA were calculated through greyscale scanning. WT RNA: wild-type HBV RNA transcripts; SP RNA: spliced HBV RNA transcripts in the rcccDNA system.

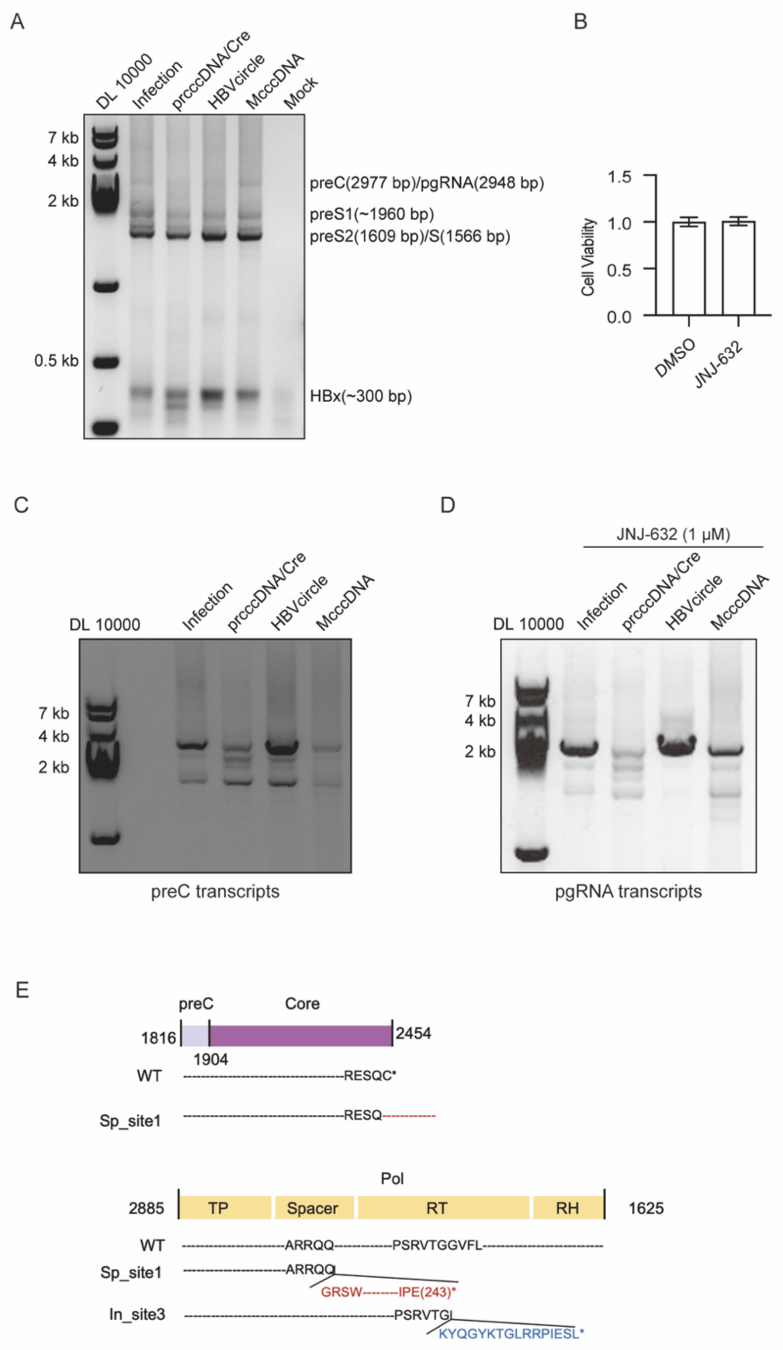

Figure S3 Amplification of major HBV transcripts in different systems by 5'RACE.

HepG2-NTCP cells were infected by HBV particles at an MOI of 300 or transfected with rcccDNA or MccDNA. Total RNA was extracted 7 days post infection or 3 days after transfection. The HBV mRNA in each group was reverse transcribed and amplified according to the indicated methods, then visualized by agarose gel electrophoresis(A). Cell viability assessment by CCK-8 assay following treatment with 1 μM JNJ-632 for three days(B). The preC-specific primer and Gsp2 primer were used to specifically amplify preC transcripts(C). Cells were treated with 1 μM JNJ-632 for 3 days and then subjected to 5'RACE. The pgRNA-specific primer and Gsp2 primer were used to specifically amplify pgRNA transcripts(D). The schematic illustrations of the protein sequences corresponding to the spliced RNA(E).

Table S1 Primer sequences used for this study.

Primers	Primer sequences (5'-3')
ccc_Seq1	ctccaacttgtcctggttatcgc
ccc_Seq2	ctccaacttgtcctggttatcgc
ccc_Seq3	ccgtctgtgccttctcatct
ccc_Seq4	gggggaggagattaggttaaagg
ccc_Seq5	ggtctcaatcgccgcgtcg
ccc_Seq6	tgtgggtcaccatattcttggg
BCP +1 fwd	catgtcctactgttcaagcctcc
BCP +1 rev	cgatacagagctgaggcggtat
P5	ctccccgtctgtgccttct
P6	gccccaaagccacccaag
SP primer 1-F	atgcccctatcctatcaacacttcc
SP primer 1-R	gtctggccaggtgtccttgt
SP primer 2-F	atccagccttcagagcaaacac
SP primer 2-R	ggtccccaatcctcgagaagattg
SP primer 3-F	agaggcctgtatttccctgct
SP primer 3-R	gacaagttggaggacaagaggttg
gapdh-s	tcgacagtcagccgcatct
gapdh-as	ctagcctcccgggtttctct
myod1-s	ccgcctgagcaaagtaaatga
myod1-as	ggcaaccgctggtttgg
GeneRacer	cgactggagcacgaggacactga
PreC Gsp	atggactgaaggagtagaaaataaattggtctgcg
Pg Gsp	actgaaggagtagaaaaactttttcacctctg
Gsp1	ggtgcgcagaccaatttatg
Gsp2	gtgcacacggtccggcagatg
MtDNA-F	taccgccatcttcagcaaac
MtDNA-R	taagggctatcgtagttttctgg
pg-2455-F	ttccttggactcataaggtgggg

pg-2871-R	gctggtggaaagattctgccc

Table S2 Antibodies used in this study.

Antibodies	Cat. NO.	Origin
Hepatitis B Surface Antigen Ab	OBT0990	Bio-rad
Hepatitis B core Antigen Ab (IF)	GB058602	Genetech
Hepatitis B core Antigen Ab (WB)		In-house
Normal Rabbit IgG	2729	CST
Anti-Histone H3 Ab	39064	Active Motif
Anti-H3ac Ab	39139	Active Motif
Anti-H4ac Ab	39925	Active Motif
Anti-H3K27ac Ab	39133	Active Motif
Anti-H3K9me3 Ab	39161	Active Motif
Anti-β-actin Ab	A2522	Sigma-Aldrich
Anti-DIG antibody	11093274910	Roche

Table S3 Sanger sequencing for McccDNA.

Primers	DNA Sequences (5'-3')
ccc_Seq1	TGCTATGCCTCATCTTCTTGTTGGTTCTTCTGGACTATCAAGGTATGTTGCC<br>CGTTTGTCCTCTAATTCCAGGATCCTCAACAACCAGCACGGGACCATGCCG<br>GACCTGCATGACTACTGCTCAAGGAACCTCTATGTATCCCTCCTGTTGCTG<br>TACCAAACCTTCGGACGGAAATTGCACCTGTATTCCCATCCCATCATCCTG<br>GGCTTTCGGAAAATTCCTATGGGAGTGGGCCTCAGCCCGTTTCTCCTGGCT<br>CAGTTTACTAGTGCCATTTGTTCAGTGGTTCGTAGGGCTTTCCCCCACTGTT<br>TGGCTTTCAGTTATATGGATGATGTGGTATTGGGGGCCAAGTCTGTACAGC<br>ATCTTGAGTCCCTTTTTACCGCTGTTACCAATTTTCTTTTGTCTTTGGGTATA<br>CATTTAAACCCTAACAAAACAAAGAGATGGGGTTACTCTCTAAATTTTATG<br>GGTTATGTCATTGGATGTTATGGGTCCTTGCCACAAGAACACATCATACAA<br>AAAATCAAAGAATGTTTTAGAAAACTTCCTATTAACAGGCCTATTGATTGG<br>AAAGTATGTCAACGAATTGTGGGTCTTTTGGGTTTTGCTGCCCCTTTTACAC<br>AATGTGGTTATCCTGCGTTGATGCCTTTGTATGCATGTATTCAATCTAAGC<br>AGGCTTTCACTTT
ccc_Seq2	TCTCGCCAACTTACAAGGCCTTTCTGTGTAAACAATACCTGAACCTTTACC<br>CCGTTGCCCGGCAACGGCCAGGTCTGTGCCAAGTGTTTGCTGACGCAACCC<br>CCACTGGCTGGGGCTTGGTCATGGGCCATCAGCGCATGCGTGGAACCTTTT<br>CGGCTCCTCTGCCGATCCATACTGCGGAACTCCTAGCCGCTTGTTTTGCTC<br>GCAGCAGGTCTGGAGCAAACATTATCGGGACTGATAACTCTGTTGTCCTAT<br>CCCGCAAATATACATCGTTTCCATGGCTGCTAGGCTGTGCTGCCAACTGGA<br>TCCTGCGCGGGACGTCCTTTGTTTACGTCCCGTCGGCGCTGAATCCTGCGG<br>ACGACCCTTCTCGGGGTCGCTTGGGACTCTCTCGTCCCCTTCTCCGTCTGCC<br>GTTCCGACCGACCACGGGGCGCACCTCTCTTTACGCGGACTCCCCGTCTGT<br>GCCTTCTCATCTGCCGGACCGTGTGCACTTCGCTTCACCTCTGCACGTCGC<br>ATGGAGACCACCGTGAACGCCCACCAAATATTGCCCAAGGTCTTACATAA<br>GAGGACTCTTGGACTCTCAGCAATGTCAACGACCGACCTTGAGGCATACTT<br>CAAAGACTGTTTGTTTAAA

ccc_Seq3	ACCACCGTGAACGCCCACCAAATATTGCCCAAGGTCTTACATAAGAGGAC<br>TCTTGGACTCTCAGCAATGTCAACGACCGACCTTGAGGCATACTTCAAAGA<br>CTGTTTGTTTAAAGACTGGGAGGAGTTGGGGGAGGAGATTAGGTTAAAGG<br>TCTTTGTACTAGGAGGCTGTAGGCATAAATTGGTCTGCGCACCAGCACCAT<br>GCAACTTTTTCACCTCTGCCTAATCATCTCTTGTTCATGTCCTACTGTTCAA<br>GCCTCCAAGCTGTGCCTTGGGTGGCTTTGGGGCATGGACATCGACCCTTAT<br>AAAGAATTTGGAGCTACTGTGGAGTTACTCTCGTTTTTGCCTTCTGACTTCT<br>TTCCTTCAGTACGAGATCTTCTAGATACCGCCTCAGCTCTGTATCGGGAAG<br>CCTTAGAGTCTCCTGAGCATTGTTCACCTCACCATACTGCACTCAGGCAAG<br>CAATTCTTTGCTGGGGGGAACTAATGACTCTAGCTACCTGGGTGGGTGTTA<br>ATTTGGAAGATCCAGCGTCTAGAGACCTAGTAGTCAGTTATGTCAACACTA<br>ATATGGGCCTAAAGTTCAGGCAACTCTTGTGGTTTCACATTTCTTGTCTCAC<br>TTTTGGAAGAGAAACAGTTATAGAGTATTTGGTGTCTTTCGGAGTGTGGAT<br>TCGCACTCCTCCAGCTT
ccc_Seq4	TGCGCACCAGCACCATGCAACTTTTTCACCTCTGCCTAATCATCTCTTGTTC<br>ATGTCCTACTGTTCAAGCCTCCAAGCTGTGCCTTGGGTGGCTTTGGGGCAT<br>GGACATCGACCCTTATAAAGAATTTGGAGCTACTGTGGAGTTACTCTCGTT<br>TTTGCCTTCTGACTTCTTTCCTTCAGTACGAGATCTTCTAGATACCGCCTCA<br>GCTCTGTATCGGGAAGCCTTAGAGTCTCCTGAGCATTGTTCACCTCACCAT<br>ACTGCACTCAGGCAAGCAATTCTTTGCTGGGGGGAACTAATGACTCTAGCT<br>ACCTGGGTGGGTGTTAATTTGGAAGATCCAGCGTCTAGAGACCTAGTAGTC<br>AGTTATGTCAACACTAATATGGGCCTAAAGTTCAGGCAACTCTTGTGGTTT<br>CACATTTCTTGTCTCACTTTTGGAAGAGAAACAGTTATAGAGTATTTGGTG<br>TCTTTCGGAGTGTGGATTCGCACTCCTCCAGCTTATAGACCACCAAATGCC<br>CCTATCCTATCAACACTTCCGGAGACTACTGTTGTTAGACGACGAGGCAGG<br>TCCCCTAGAAGAAGAACTCCCTCGCCTCGCAGACGAAGGTCTCAATCGCC<br>GCGTCGCAGAAGATCTCAATCTCGGGAATCTCAATGTTAGTATTCCTTGGA<br>CTCATAAGGTGGGGAAC
ccc_Seq5	CATAAGGTGGGGAACTTTACTGGGCTTTATTCTTCTACTGTACCTGTCTTTA<br>ATCCTCATTGGAAAACACCATCTTTTCCTAATATACATTTACACCAAGACA<br>TTATCAAAAAATGTGAACAGTTTGTAGGCCCACTCACAGTTAATGAGAAA<br>AGAAGATTGCAATTGATTATGCCTGCCAGGTTTTATCCAAAGGTTACCAAA<br>TATTTACCATTGGATAAGGGTATTAAACCTTATTATCCAGAACATCTAGTT<br>AATCATTACTTCCAAACTAGACACTATTTACACACTCTATGGAAGGCGGGT<br>ATATTATATAAGAGAGAAACAACACATAGCGCCTCATTTTGTGGGTCACC<br>ATATTCTTGGGAACAAGATCTACAGCATGGGGCAGAATCTTTCCACCAGC<br>AATCCTCTGGGATTCTTTCCCGACCACCAGTTGGATCCAGCCTTCAGAGCA<br>AACACCGCAAATCCAGATTGGGACTTCAATCCCAACAAGGACACCTGGCC<br>AGACGCCAACAAGGTAGGAGCTGGAGCATTCGGGCTGGGTTTCACCCCAC<br>CGCACGGAGGCCTTTTGGGGTGGAGCCCTCAGGCTCAGGGCATACTACAA<br>ACTTTGCCAGCAAATCCGCCTCCTGCCTCCACCAATCGCCAGTCAGGAAGG<br>CAGCCTACCCCGCTGTCTCCACC

ccc_Seq6	CTCTGGGATTCTTTCCCGACCACCAGTTGGATCCAGCCTTCAGAGCAAACA<br>CCGCAAATCCAGATTGGGACTTCAATCCCAACAAGGACACCTGGCCAGAC<br>GCCAACAAGGTAGGAGCTGGAGCATTCGGGCTGGGTTTCACCCCACCGCA<br>CGGAGGCCTTTTGGGGTGGAGCCCTCAGGCTCAGGGCATACTACAAACTTT<br>GCCAGCAAATCCGCCTCCTGCCTCCACCAATCGCCAGTCAGGAAGGCAGC<br>CTACCCCGCTGTCTCCACCTTTGAGAAACACTCATCCTCAGGCCATGCAGT<br>GGAATTCCACAACCTTCCACCAAACTCTGCAAGATCCCAGAGTGAGAGGC<br>CTGTATTTCCCTGCTGGTGGCTCCAGTTCAGGAACAGTAAACCCTGTTCTG<br>ACTACTGCCTCTCCCTTATCGTCAATCTTCTCGAGGATTGGGGACCCTGCG<br>CTGAACATGGAGAACATCACATCAGGATTCCTAGGACCCCTTCTCGTGTTA<br>CAGGCGGGGTTTTTCTTGTTGACAAGAATCCTCACAATACCGCAGAGTCTA<br>GACTCGTGGTGGACTTCTCTCAATTTTCTAGGGGGAACTACCGTGTGTCTT<br>GGCCAAAATTCGCAGTCCCCAACCTCCAATCACTCACCAACCTCTTGTCCT<br>CCAACTTGTCCTGGTTATCGCTGGATGTGTCTGCGGCGTTTTATCATCTTCC<br>TCTTCATCCTGCTGCTATGCCTCATCTTCTTGTTGGTTCTTCTGGACTATCA<br>AGGTATGTTGCCCGTTT
